# Clinical characteristics of sarcopenia in patients with alcoholic liver cirrhosis

**DOI:** 10.1002/jgh3.12582

**Published:** 2021-05-29

**Authors:** Chisato Saeki, Tomoya Kanai, Masanori Nakano, Tsunekazu Oikawa, Yuichi Torisu, Masayuki Saruta, Akihito Tsubota

**Affiliations:** ^1^ Division of Gastroenterology and Hepatology, Department of Internal Medicine The Jikei University School of Medicine Tokyo Japan; ^2^ Division of Gastroenterology, Department of Internal Medicine Fuji City General Hospital Shizuoka Japan; ^3^ Core Research Facilities, Research Center for Medical Science The Jikei University School of Medicine Tokyo Japan

**Keywords:** alcohol consumption, alcoholic liver cirrhosis, sarcopenia

## Abstract

**Background and Aim:**

Sarcopenia frequently develops in patient with liver cirrhosis (LC). Ethanol reduces muscle protein synthesis and accelerates proteolysis. However, the relationship between heavy alcohol consumption and sarcopenia remains controversial. This study aimed to investigate the characteristics and prevalence of sarcopenia among patients with alcoholic LC (ALC) in real‐world clinical settings.

**Methods:**

This cross‐sectional study included 181 patients with LC. Heavy alcohol consumption was defined as >60 g/day. Sarcopenia was diagnosed according to the Japan Society of Hepatology criteria.

**Results:**

Among the 181 patients, 64 (35.4%) were diagnosed with ALC. Patients with ALC were younger (median, 61.5 *vs* 72.0 years; *P* < 0.001) and had a lower prevalence of sarcopenia (18.8 *vs* 32.5%; *P* = 0.048) than those with non‐ALC. Conversely, the former had a higher prevalence of Child–Pugh class B/C (*P* = 0.015), higher total bilirubin (*P* = 0.017), and lower prothrombin time (*P* < 0.001) than the latter. The prevalence of sarcopenia increased alongside advancing age in patients with ALC (*P* = 0.007). Multivariate analysis identified older age (but not disease stage/liver function reserve and alcohol consumption) as an independent factor associated with sarcopenia (*P* = 0.002) in patients with ALC.

**Conclusion:**

Patients with ALC were younger and had a lower prevalence of sarcopenia, despite advanced disease stage/impaired liver function reserve, compared to those with non‐ALC in real‐world clinical settings. However, older age was strongly associated with sarcopenia, even in patients with ALC. There was no significant influence of heavy alcohol consumption on the development of sarcopenia.

## Graphical abstract

This study aimed to investigate the characteristics and prevalence of sarcopenia among 181 patients with alcoholic liver cirrhosis (ALC). Patients with ALC were younger and had a lower prevalence of sarcopenia, despite advanced disease stage/impaired liver function reserve, compared to those with non‐ALC.

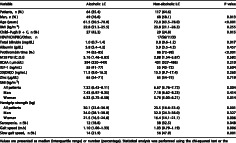

## Introduction

Alcoholic liver disease (ALD) comprises a wide spectrum of liver diseases ranging from alcoholic hepatitis to steatosis, steatohepatitis, fibrosis, and liver cirrhosis (LC), and is one of the most common causes of liver disease worldwide.^1^, ^2^ Heavy alcohol consumption is responsible for high mortality and burden of disease.^1^, ^2^ According to the World Health Organization reports in 2016, harmful alcohol consumption resulted in approximately 3 million deaths (5.3% of all deaths), and 132 million disability‐adjusted life years (DALYs) (5.1% of all DALYs) worldwide.^1^ Conversely, over 50% of mortality due to LC is attributable to harmful alcohol consumption worldwide.^3^, ^4^ Therefore, alcohol consumption and alcohol‐related health conditions are a global concern, and appropriate management of ALD is crucial, especially in patients with LC.

Protein‐energy malnutrition (PEM) is a common complication of ALD and causes sarcopenia, which is characterized by a generalized loss of skeletal muscle mass and strength.^5^, ^6^, ^7^ Ethanol impairs the mammalian target of rapamycin (mTOR) signaling pathway and decreases muscle protein synthesis predominately in type II muscle fibers.^6^, ^7^, ^8^ Furthermore, ethanol‐induced protein degradation and resultant muscle loss are manifested via skeletal muscle autophagy.^7^, ^9^, ^10^ Indeed, heavy alcohol consumption is associated with sarcopenia in elderly patients with liver disease.^11^ However, in the general population without liver disease, alcohol consumption does not increase the risk of sarcopenia because heavy drinkers are younger and exercise more consistently than non‐heavy drinkers.^11^ Additionally, a meta‐analysis of 13 studies demonstrated that alcohol consumption is not a risk factor for sarcopenia.^12^ Therefore, the relationship between heavy alcohol consumption and sarcopenia remains controversial.

The European Working Group on Sarcopenia in Older People (EWGSOP) classified sarcopenia into two categories: primary, when sarcopenia is related to aging; and secondary, when one or more other causes are present; chronic diseases, including chronic liver disease (CLD).^13^ The current sarcopenia criteria for patients with CLD (criteria for secondary sarcopenia) proposed by the Japan Society of Hepatology (JSH) do not include an age‐related criterion because sarcopenia in CLD is predominantly secondary to disease or nutrition‐related problems and can occur in non‐elderly patients.^14^ Indeed, the prevalence of sarcopenia demonstrates a linear increase with the progression of disease in patients with CLD.^14^, ^15^, ^16^

In the present study, we aimed at investigating the characteristics and prevalence of sarcopenia in patients with alcoholic LC (ALC) in real‐world clinical settings (including non‐elderly patients).

## Methods

### Study design and patients

This cross‐sectional study included 181 patients with LC who presented to the Jikei University School of Medicine (Tokyo, Japan) and Fuji City General Hospital (Shizuoka, Japan) between February 2017 and November 2020. The inclusion criteria were as follows: (i) LC due to any etiology; (ii) available data on skeletal muscle mass index (SMI) evaluated using bioelectrical impedance analysis (BIA) (InBody S10; InBody, Seoul, Korea) and grip strength using a dynamometer (T.K.K5401 GRIP‐D; Takei Scientific Instruments, Niigata, Japan); and (iii) available data on alcohol consumption history in the medical records. LC was diagnosed based on laboratory tests, morphological findings on imaging (ultrasonography, computed tomography, and/or magnetic resonance), and presentation of portal hypertension (such as esophageal/gastric varices and ascites). ALC was diagnosed based on LC with current and/or past history of heavy alcohol consumption (>60 g/day) and exclusion of other etiologies, such as hepatitis B or C, autoimmune hepatitis, primary biliary cholangitis, and non‐alcoholic steatohepatitis.^17^ Current drinking was defined as continuous heavy alcohol consumption at least within the previous month preceding the survey. Patients with refractory ascites, implants, or hemodialysis were excluded.^18^ This study was approved by the Ethics Committee of the Jikei University School of Medicine (approval no. 28‐196) and Fuji City General Hospital (approval No. 156) and conducted in accordance with the Declaration of Helsinki. Written informed consent was obtained from all the participants.

### Diagnosis of sarcopenia and slow gait speed

Sarcopenia was diagnosed based on the criteria established by the JSH.^14^ Sarcopenia was defined as reduced handgrip strength (<26 kg in men and <18 kg in women) and muscle mass (SMI <7.0 kg/m^2^ in men and <5.7 kg/m^2^ in women). Additionally, gait speed was assessed over 6 m. Slow gait speed was defined as a speed <1.0 m/s.

### Laboratory assessments

The following serum parameters were evaluated using standard laboratory methods: total bilirubin, albumin, mac‐2 binding protein glycosylation isomer (M2BPGi; hepatic fibrosis marker), branched‐chain amino acid (BCAA), insulin‐like growth factor‐1 (IGF‐1), 25‐hydroxyvitamin D (25(OH)D), zinc, and prothrombin time (PT).

### Statistical analysis

Continuous and categorical variables were presented as medians (interquartile ranges) and numbers (percentages), respectively. The difference between the groups was evaluated using the Mann–Whitney *U* test or the Kruskal–Wallis test followed by the Steel–Dwass post hoc test for continuous variables and chi‐squared test for categorical variables. The Cochran–Armitage trend test was performed to analyze whether a trend was present between a variable with two categories and a variable with multiple categories. Univariate and multiple logistic regression analyses were performed to identify significant and independent factors associated with sarcopenia. All statistical analyses were performed using SPSS (IBM Japan, Tokyo, Japan). A *P* value <0.05 was considered statistically significant.

## Results

### Comparison of clinical characteristics between patients with and without ALC

Among the 181 patients with LC, 64 (35.4%) were diagnosed with ALC (Table 1). Patients with ALC (ALC group) were significantly younger than those in the non‐ALC group (*P* < 0.001). The ratios of male patients (*P* = 0.013) and Child–Pugh class B/C (i.e. decompensated LC; *P* = 0.015) were significantly higher in the ALC group than in the non‐ALC group. Regarding the biochemical parameters, the ALC group had significantly higher total bilirubin (*P* = 0.017) and lower PT levels (*P* < 0.001) than the non‐ALC group. SMI and handgrip strength values were higher in the ALC group than in the non‐ALC group, although the significance differed between female and male patients. The ALC group had a significantly lower prevalence of sarcopenia (18.8 *vs* 32.5%; *P* = 0.048) and slow gate speed (21.9 *vs* 47.9%; *P* = 0.001) than the non‐ALC group. Taken together, despite more advanced liver disease or impaired liver function reserve, patients in the ALC group were younger, and had a lower prevalence of sarcopenia (higher SMI and handgrip strength values) and slow gait speed (higher gait speed) than those in the non‐ALC group.

**Table 1:** Comparison of clinical characteristics between patients with and without alcoholic liver cirrhosis

Variable	Alcoholic LC	Non‐alcoholic LC	*P* value
Patients, *n* (%)	64 (35.4)	117 (64.6)	
Man, *n* (%)	49 (76.6)	68 (58.1)	0.013
Age (years)	61.5 (50.5–70.0)	72.0 (63.5–78.0)	<0.001
BMI (kg/m^2^)	22.8 (21.2–25.5)	23.8 (21.1–26.3)	0.255
Child–Pugh B + C, *n* (%)	27 (42.2)	29 (24.8)	0.015
HBV/HCV/PBC/Other, *n*		17/56/11/33	
Total bilirubin (mg/dL)	1.0 (0.7–1.4)	0.8 (0.6–1.2)	0.017
Albumin (g/dL)	3.8 (3.4–4.2)	3.9 (3.5–4.3)	0.457
Prothrombin time (%)	74 (62–85)	86 (72–98)	<0.001
M2BPGi (C.O.I)	3.16 (1.46–6.82)	2.88 (1.54–5.87)	0.582
BCAA (μmol/L)	394 (320–449)	401 (327–486)	0.210
IGF‐1 (ng/mL)	55 (41–77)	55 (43–72)	0.604
25(OH)D (ng/mL)	11.5 (9.0–16.2)	13.3 (9.7–17.4)	0.265
Zinc (μg/dL)	65 (54–71)	65 (54–75)	0.719
SMI (kg/m^2^)			
All patients	7.32 (6.43–8.11)	6.67 (5.76–7.72)	0.004
Man	7.43 (6.97–8.35)	7.18 (6.62–8.23)	0.414
Woman	6.33 (5.75–6.58)	5.76 (5.03–6.21)	0.014
Handgrip strength (kg)			
All patients	30.1 (23.6–36.9)	23.5 (16.6–33.4)	0.001
Man	34.0 (28.1–38.8)	32.0 (24.3–38.0)	0.327
Woman	21.5 (16.5–24.6)	16.4 (14.1–21.1)	0.006
Sarcopenia, *n* (%)	12 (18.8)	38 (32.5)	0.048
Gait speed (m/s)	1.10 (1.00–1.33)	1.03 (0.79–1.19)	0.006
Slow gait speed, *n* (%)	14 (21.9)	56 (47.9)	0.001

Values are presented as median (interquartile range) or number (percentage). Statistical analysis was performed using the chi‐squared test or the Mann–Whitney *U* test, as appropriate.

25(OH)D, 25‐hydroxyvitamin D; BCAA, branched‐chain amino acid; BMI, body mass index; HBV, hepatitis B virus; HCV, hepatitis C virus; IGF‐1, insulin‐like growth factor 1; LC, liver cirrhosis; M2BPGi, Mac‐2 binding protein glycosylation isomer; PBC, primary biliary cholangitis; SMI, skeletal muscle mass index.

### Proportion of ALC and non‐ALC in the age groups

**Figure 1:**
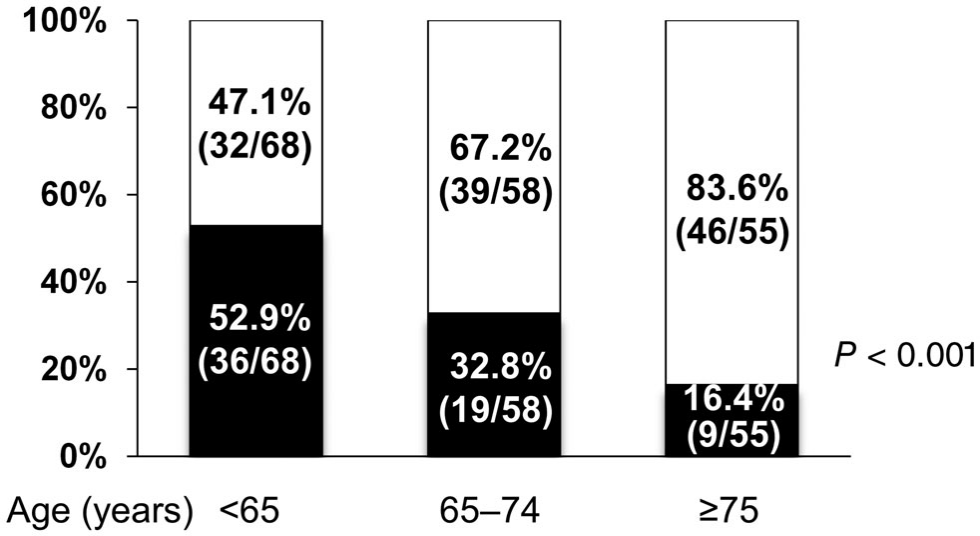
The proportion of alcoholic liver cirrhosis (ALC) and non‐ALC among the three age groups (<65, 65–74, and ≥75 years). The proportion of ALC significantly decreased stepwise with advancing age, whereas the proportion of non‐ALC significantly increased (*P* < 0.001 by the Cochran–Armitage trend test). (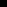), ALC; (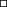), non‐ALC.

Since the patients in the ALC group were younger than those in the non‐ALC group, we stratified the patients into three groups according to age (<65, 65–74, and ≥75 years) and investigated the proportion of patients with ALC and non‐ALC in each age group (Fig. 1). The proportion of patients with ALC significantly decreased alongside advancing age, whereas the proportion of patients with non‐ALC significantly increased (both *P* < 0.001).

**Figure 2:**
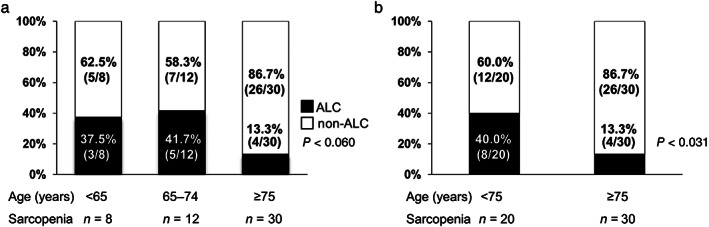
(a) The proportion of alcoholic liver cirrhosis (ALC) and non‐ALC in the three age groups among patients with sarcopenia (<65, 65–74, and ≥75 years). There was a marginally significant difference among the three groups (*P* = 0.060 by the Cochran–Armitage trend test). (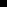), ALC; (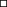), non‐ALC. (b) The proportion of ALC and non‐ALC in the two age groups among patients with sarcopenia (<75 and ≥75 years). The proportion of ALC significantly decreased stepwise with advancing age, whereas the proportion of non‐ALC significantly increased (*P* = 0.031 by the Cochran–Armitage trend test). (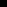), ALC; (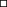), non‐ALC.

Regarding the proportion of patients with ALC and non‐ALC in each age group among patients with sarcopenia, there was a marginally significant trend among the three groups (*P* = 0.060; Fig. 2a). When the patients were re‐stratified into two groups (<75 *vs* ≥75 years), the proportion of patients with ALC significantly decreased in the advanced age group (*P* = 0.031; Fig. 2b).

### Comparison between sarcopenia and non‐sarcopenia in the ALC group

Table 2 displays the characteristics of patients with sarcopenia in the ALC group. The cumulative alcohol consumption and prevalence of current drinking did not significantly differ between patient with and without sarcopenia. Patients with sarcopenia were older (*P* = 0.020) and had lower body mass index (BMI; *P* = 0.006) and serum BCAA levels (*P* = 0.006) than those without sarcopenia. Moreover, the former had lower gait speed (*P* < 0.001) and a higher prevalence of slow gait speed than the latter (75.0 *vs* 9.6%; *P* < 0.001).

**Table 2:** Comparison of clinical characteristics between patients with and without sarcopenia in patients with alcoholic liver cirrhosis

Variable	Sarcopenia	Non‐sarcopenia	*P* value
Patients, *n* (%)	12 (18.8)	52 (81.3)	
Man, *n* (%)	10 (83.3)	39 (75.0)	0.539
Age (years)	73.5 (59.0–75.0)	59.0 (50.0–68.0)	0.020
BMI (kg/m^2^)	21.9 (18.8–22.3)	23.6 (21.5–26.2)	0.006
Cumulative alcohol consumption (kg)	1095 (908–1256)	1026 (770–1311)	0.667
Current drinking, *n* (%)	7 (58.3)	27 (51.9)	0.688
Child–Pugh B + C, *n* (%)	5 (41.7)	22 (42.3)	0.968
Total bilirubin (mg/dL)	1.0 (0.7–1.7)	1.0 (0.7–1.4)	0.829
Albumin (g/dL)	3.5 (3.4–4.0)	3.9 (3.4–4.3)	0.215
Prothrombin time (%)	76 (66–91)	74 (62–85)	0.486
M2BPGi (C.O.I)	2.68 (1.95–8.17)	3.39 (1.39–6.73)	0.730
BCAA (μmol/L)	336 (231–350)	407 (336–478)	0.006
IGF‐1 (ng/mL)	49 (34–63)	59 (41–82)	0.156
25(OH)D (ng/mL)	10.1 (9.1–17.0)	11.9 (8.9–16.2)	0.919
Zinc (μg/dL)	58 (47–69)	65 (58–73)	0.146
SMI (kg/m^2^)	6.14 (5.56–6.95)	7.42 (6.91–8.32)	<0.001
Handgrip strength (kg)	20.0 (16.4–25.0)	32.8 (28.2–38.4)	<0.001
Gait speed (m/s)	0.84 (0.62–1.04)	1.23 (1.03–1.35)	<0.001
Slow gait speed, *n* (%)	9 (75.0)	5 (9.6)	<0.001

Values are presented as median (interquartile range) or number (percentage). Statistical analysis was performed using the chi‐squared test or the Mann–Whitney *U* test, as appropriate.

25(OH)D, 25‐hydroxyvitamin D; BCAA, branched‐chain amino acid; BMI, body mass index; IGF‐1, insulin‐like growth factor 1; M2BPGi, Mac‐2 binding protein glycosylation isomer; SMI, skeletal muscle mass index.

### Factors related to sarcopenia in the ALC group

In univariate analyses, the following three variables were significantly associated with sarcopenia: age, BMI, and serum BCAA levels (Table S1). In the multivariate analysis, the following two variables were significant and independent (Table 3): older age (odds ratio [OR], 1.218; 95% confidence interval [CI], 1.073–1.384; *P* = 0.002) and lower serum BCAA levels (OR, 0.971; 95% CI, 0.954–0.989; *P* = 0.002).

**Table 3:** Factors associated with sarcopenia in patients with alcoholic liver cirrhosis

	Univariate		Multivariate	
Variable	OR (95% CI)	*P* value	OR (95% CI)	*P* value
Age (years)	1.075 (1.009–1.147)	0.026	1.218 (1.073–1.384)	0.002
BMI (kg/m^2^)	0.696 (0.522–0.928)	0.013		
BCAA (μmol/L)	0.988 (0.980–0.997)	0.007	0.971 (0.954–0.989)	0.002

BCAA, branched‐chain amino acid; BMI, body mass index; CI, confidence interval; OR, odds ratio.

### Clinical characteristics of the three age groups in the ALC group

**Figure 3:**
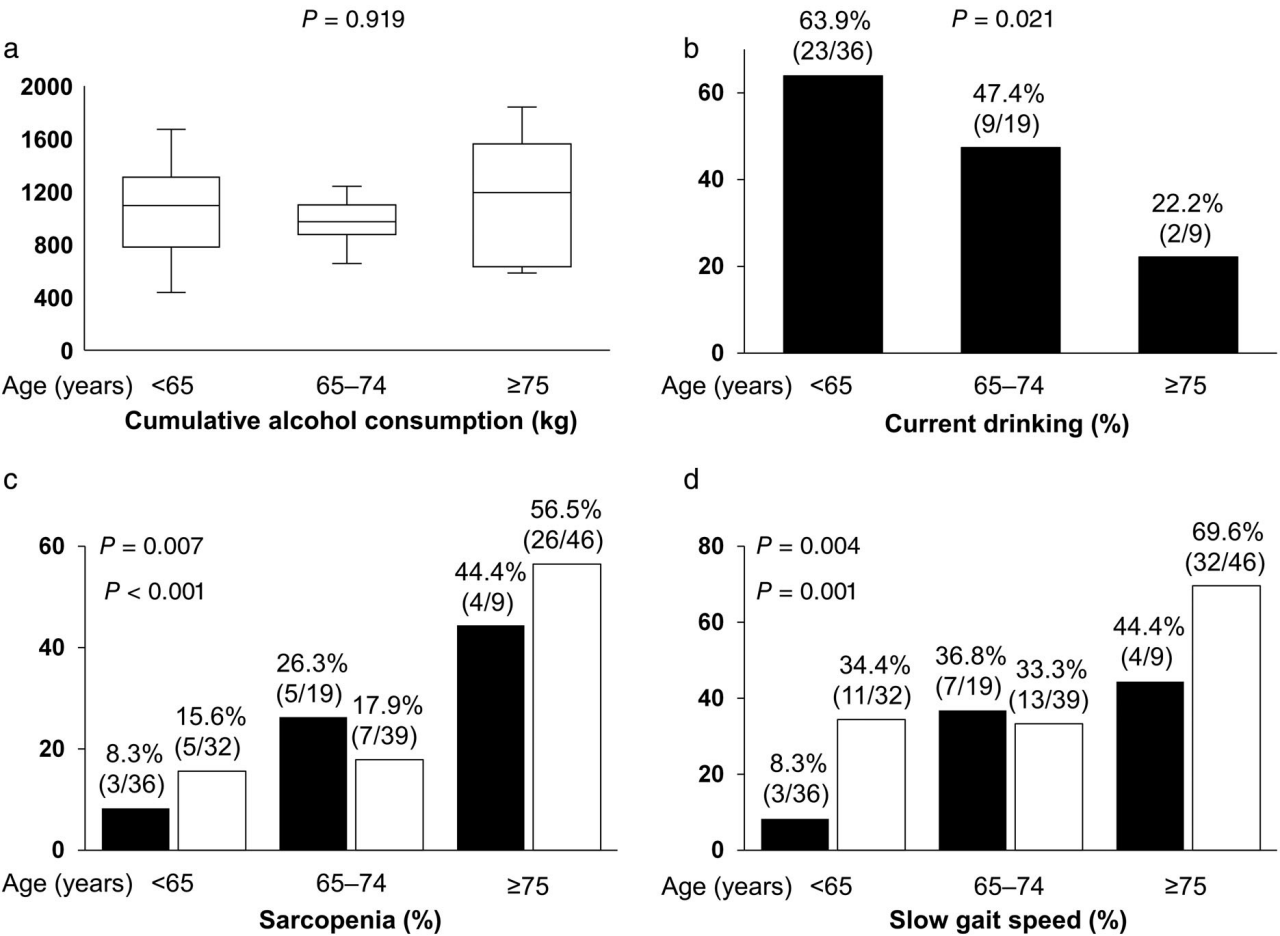
Comparison of alcohol consumption situations and the prevalence of sarcopenia and slow gait speed among the three age groups (<65, 65–74, and ≥75 years). (a) Cumulative alcohol consumption did not significantly differ among the three groups in the alcoholic liver cirrhosis (ALC) group (*P* = 0.919 by the Kruskal–Wallis test). (b) The prevalence of current drinking significantly decreased stepwise with advancing age in the ALC group (*P* = 0.021 by the Cochran–Armitage trend test). (c), (d) The prevalence of sarcopenia and slow gait speed increased stepwise with advancing age in the ALC group (*P* = 0.007 and 0.004, respectively, by the Cochran–Armitage trend test) and non‐ALC group (*P* < 0.001 and 0.001, respectively, by the Cochran–Armitage trend test). (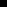), ALC; (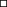), non‐ALC.

As described above, the patients in the ALC group were younger and had a lower prevalence of sarcopenia than those in the non‐ALC group. However, when limited to the ALC group, older age was significantly and independently associated with sarcopenia. Therefore, we stratified the patients with ALC into three groups according to age (<65, 65–74, and ≥75 years) and compared the alcohol consumption situation and the prevalence of sarcopenia and slow gait speed (Fig. 3). The cumulative alcohol consumption did not significantly differ among the three groups (*P* = 0.919; Fig. 3a). However, the prevalence of current drinking significantly decreased stepwise with advancing age (*P* = 0.021; Fig. 3b).

Similarly, the patients with non‐ALC were stratified into three age groups. The prevalence of sarcopenia significantly increased stepwise with advancing age in both the ALC and non‐ALC groups (*P* = 0.007 and <0.001, respectively; Fig. 3c). The prevalence of slow gait speed also significantly increased stepwise with advancing age in both groups (*P* = 0.004 and 0.001, respectively; Fig. 3d).

## Discussion

Alcohol abuse and its effect on health are global concerns, given that harmful alcohol use is associated with mortality, quality of life, and life‐threatening diseases (including LC).^1^, ^2^, ^5^ Heavy alcohol consumption causes PEM, impairs muscle protein synthesis, and accelerates proteolysis, thereby resulting in sarcopenia.^5^ Many studies have investigated malnutrition and sarcopenia (as differently defined by different groups) in patients with ALD.^6^, ^7^, ^19^ However, the relationship between alcohol consumption and sarcopenia remains controversial. To the best of our knowledge, this is the first report to specify the clinical characteristics of sarcopenia (as defined by the JSH criteria) in patients with ALC, comparing them in patients with non‐ALC in real‐world clinical settings (including non‐elderly patients).

The prevalence of sarcopenia increases linearly with the progression of CLD to advanced disease stages.^14^, ^15^, ^16^ Specifically, sarcopenia commonly develops in patients with decompensated LC.^16^ However, in the present study, the ALC group with more advanced disease stage or impaired liver function reserve had a lower prevalence of sarcopenia and slow gait speed than the non‐ALC group. These paradoxical findings may arise from clinical characteristics peculiar to the ALC group: (i) the ALC group was 10 years younger in median age than the non‐ALC group; (ii) the prevalence of current drinking (but not cumulative alcohol consumption) was higher for younger patients and this may severely worsen liver function and further advance the disease stage; and (iii) older age (but not more advanced disease stage/impaired liver function reserve and current drinking) was involved in the development of sarcopenia even in the relatively young ALC group.

In one study of elderly men aged ≥65 years among the general population, heavy drinkers had higher SMI than non‐heavy drinkers.^11^ One possible explanation for this finding may be that heavy drinkers were younger and exercised more consistently. However, when limited to subjects with liver dysfunction and adjusted for confounding factors, heavy alcohol consumption was related to lower SMI.^11^ From another viewpoint, our present study clarified the relationship among age, sarcopenia, and alcohol consumption in patients with ALC: older age (but not cumulative alcohol consumption or current drinking) was significantly and independently associated with sarcopenia, and the prevalence of sarcopenia significantly increased alongside advancing age. In another study of patients with LC, patients with ALD were younger than those with chronic hepatitis C and non‐alcoholic fatty liver disease (56, 67, and 63 years, respectively) and had a higher prevalence of decompensated LC (48, 8, and 17%, respectively) and poorer prognosis.^20^ These findings suggest that patients with ALC are younger and may be more physically active and, therefore, have a lower prevalence of sarcopenia and slow gait speed, despite more advanced disease stage/impaired liver function reserve. In the present study, there was no influence of alcohol consumption on the development of sarcopenia.

Furthermore, patients with ALC seem to have different characteristics from those with LC caused by other etiologies. In general, advanced liver disease can cause secondary sarcopenia.^14^, ^15^, ^16^ However, younger patients with ALC did not have sarcopenia despite more advanced disease stage/impaired liver function reserve. Patients with ALC who continue heavy alcohol consumption may not live longer due to an earlier onset of fatal complications; variceal rupture, liver failure, and bacterial infection, compared to those with non‐ALC. Therefore, the proportion of patients with ALC and sarcopenia might decrease with advancing age in the present study cohort. It may be more advisable to treat patients with ALC separately from those with LC caused by other etiologies in the analysis of sarcopenia.

In the present study, the serum BCAA level was a significant, independent factor of sarcopenia in patients with ALC. Skeletal muscle mass is maintained by a balance between protein synthesis and proteolysis.^7^ BCAAs are involved in muscle protein synthesis and activation of satellite cells (precursors to new muscle fibers) through the mTOR signaling pathway.^21^ PEM and hyperammonemia cause the consumption of BCAAs in the skeletal muscles for energy production and ammonia detoxification in patients with LC, which further leads to exacerbation of sarcopenia.^22^, ^23^ Ethanol directly reduces muscle protein synthesis by impairing the mTOR signaling pathway.^6^, ^7^, ^8^ Moreover, ethanol and its metabolites induce muscle autophagy, thereby leading to muscle protein breakdown.^7^, ^9^, ^10^ Leucine‐enriched BCAA supplementation restores impaired mTOR signaling and increased autophagy in patients with ALC.^10^ These findings suggest that reduced BCAA levels and increased ethanol consumption collaboratively break proteostasis and consequently induce sarcopenia. Therefore, abstinence from alcohol and nutritional interventions (including BCAA supplementation) are crucial in patients with ALC.

This study has some limitations. First, we did not investigate the daily physical exercise level and nutritional intake. Second, we did not include age‐ and sex‐matched controls because patients with ALC were apparently younger and the ratio of male to female was higher among patients with ALC than that among those with LC caused by other etiologies. Third, the sample size was not large enough to determine the characteristics and factors related to sarcopenia in patients with ALC. Fourth, the BIA system used for evaluating skeletal muscle mass has a critical limitation in some patients with decompensated LC in that it can overestimate the measurements in the presence of some patient conditions, such as ascites and edema.^24^ Finally, we did not investigate longitudinal morbidity and mortality in patients with and without sarcopenia because this was a cross‐sectional study.

In conclusion, in the present study, we demonstrated that patients with ALC were significantly younger and had a lower prevalence of sarcopenia and slow gait speed, despite more advanced disease stage/impaired liver function reserve, compared to those with non‐ALC in the real‐world clinical settings. Therefore, it may be more advisable to treat patients with ALC separately from those with LC caused by other etiologies in the analysis of sarcopenia. However, older age was strongly associated with sarcopenia, even in patients with ALC. Finally, there was no significant influence of heavy alcohol consumption on the development of sarcopenia in our study.

## Supporting information


**Table S1.** Univariate analysis for factors associated with sarcopenia in patients with alcoholic liver cirrhosis.
