# Supplementary material for: Clinical characteristics of sarcopenia in patients with alcoholic liver cirrhosis
Source: JGH Open. 2021 May 29;5(7):763–9. doi: 10.1002/jgh3.12582 (PMC8264247; doi:10.1002/jgh3.12582)
Supplement: Supplementary file 1 — Table S1. Univariate analysis for factors associated with sarcopenia in patients with alcoholic liver cirrhosis. [file JGH3-5-763-s001.docx]

**Table S1. Univariate analysis for factors associated with sarcopenia in patients with alcoholic liver cirrhosis**

| Variable | OR (95% CI) | *p* value |
| --- | --- | --- |
| Gender (Man) | 1.667 (0.322–8.616) | 0.542 |
| Age (years) | 1.075 (1.009–1.147) | 0.026 |
| BMI (kg/m^2^) | 0.696 (0.522–0.928) | 0.013 |
| Cumulative alcohol  consumption (kg) | 1.000 (0.999–1.001) | 0.999 |
| Current drinking | 1.296 (0.364–4.616) | 0.689 |
| Child-Pugh B+C | 0.974 (0.273–3.478) | 0.968 |
| Total bilirubin (mg/dL) | 1.010 (0.389–2.623) | 0.983 |
| Albumin (g/dL) | 0.460 (0.139–1.521) | 0.203 |
| Prothrombin time (%) | 1.017 (0.974–1.061) | 0.444 |
| M2BPGi (C.O.I) | 1.032 (0.868–1.227) | 0.721 |
| BCAA (μmol/L) | 0.988 (0.980–0.997) | 0.007 |
| IGF-1 (ng/mL) | 0.976 (0.948–1.006) | 0.117 |
| 25(OH)D (ng/mL) | 1.043 (0.964–1.128) | 0.294 |
| Zinc (µg/dL) | 0.980 (0.937–1.026) | 0.386 |

25(OH)D, 25-hydroxyvitamin D; BCAA, branched-chain amino acid; BMI, body mass index; CI, confidence interval; IGF-1, insulin-like growth factor 1; M2BPGi, Mac-2 binding protein glycosylation isomer; OR, odds ratio.
